# Error Model and Compensation of Bell-Shaped Vibratory Gyro

**DOI:** 10.3390/s150923684

**Published:** 2015-09-17

**Authors:** Zhong Su, Ning Liu, Qing Li

**Affiliations:** 1 School of Automation, Beijing Institute of Technology, Beijing 100081, China; E-Mail: liuning1898@bit.edu.cn; 2 Beijing Key Laboratory of High Dynamic Navigation Technology, Beijing Information Science & Technological University, Beijing 100101, China; E-Mail: liqing@bistu.edu.cn

**Keywords:** Coriolis vibratory gyro, error model, error compensation, bell-shaped vibratory gyro

## Abstract

A bell-shaped vibratory angular velocity gyro (BVG), inspired by the Chinese traditional bell, is a type of axisymmetric shell resonator gyroscope. This paper focuses on development of an error model and compensation of the BVG. A dynamic equation is firstly established, based on a study of the BVG working mechanism. This equation is then used to evaluate the relationship between the angular rate output signal and bell-shaped resonator character, analyze the influence of the main error sources and set up an error model for the BVG. The error sources are classified from the error propagation characteristics, and the compensation method is presented based on the error model. Finally, using the error model and compensation method, the BVG is calibrated experimentally including rough compensation, temperature and bias compensation, scale factor compensation and noise filter. The experimentally obtained bias instability is from 20.5°/h to 4.7°/h, the random walk is from 2.8°/h^1/2^ to 0.7°/h^1/2^ and the nonlinearity is from 0.2% to 0.03%. Based on the error compensation, it is shown that there is a good linear relationship between the sensing signal and the angular velocity, suggesting that the BVG is a good candidate for the field of low and medium rotational speed measurement.

## 1. Introduction

The vibratory gyroscope is a specialized branch of the gyro research field, which is gaining more attention from researchers [1,2]. The core component of the vibratory gyro is a micro mechanical structure which works in the resonant state and is therefore called the resonator. While the resonator rotates around the sensitive axis, the Coriolis force induces movement of the resonator’s mode shape. There are several different types of resonator available including fork, beam, finger, axisymmetric shell *etc.* The axisymmetric shell resonator gyroscope currently gives the best comprehensive performance and is widely applied in many fields [3,4]. The cylindrical resonator and hemispherical resonator both use a single curve surface shell. These shells have the disadvantage of vibration instability. The development tendency is towards micro and multi curved surface [5].

The bell-shaped vibratory gyro is a solid vibratory gyro, which utilizes the standing wave precession effect for sensory control of the angular velocity. The core component is a millimeter Chinese traditional bell which is named the bell-shaped resonator. The excitation and detection piezoelectric elements attach to the wall of the resonator, and control the resonator to produce the standing wave. The precession of the standing wave is proportional to the angular velocity. BVG not only has the advantages of low cost, low power consumption and longevity, but is also a simple structure with good anti-impact performance, which is well suited to the low and medium rotation angular measurement fields [6,7,8]. In [7,8], analysis, design and experiments for a BVG prototype are presented and the character of the bell-shaped resonator is studied which has isolating holes. The holes are designed to isolate the vibration from the fixation point. But the positions of the holes have an important impact on the structure, especially on the frequency split. This is why holes have been removed in this paper. In [9], the signal process method of BVG is presented, which is verified experimentally in a laboratory environment at room temperature. In [10], the disadvantages of traditional BVG signal processing are addressed by presenting a novel signal processing method using a variable structure sliding mode controller to evaluate the angular velocity. This method evidently improves the accuracy and bandwidth of the BVG. In [11,12], the effects of frequency split on error are studied and a restraint method is presented based on structure balance and circuit control.

In conclusion, the BVG is still in the prototype development phase. The mathematic model, signal processing, standing wave characteristics and frequency splitting were studied. There is an urgent requirement to improve the performance of the BVG through error compensation.

The hemispherical resonator gyro (HRG) and cylinder vibratory gyro (CVG) is currently the focus for many researchers. There has already been a lot of study on their error characteristics. V. A. Matveev wrote a book on the Solid Vibratory Gyro, which studies mainly the HRG including the mathematic model, signal processing method, error characteristics and application. This book describes a problem in the HRG development process. This research laid the foundation for a further performance study of HRG [4]. J. Pi has designed an error compensation method based on an imperfect observer to restrain the drift of HRG through the state space. The observer can compensate for errors using the imperfect model parameter [13]. This paper focuses on improving the accuracy of this method using a control method during the signal solving process, but the error compensation for HRG is not researched. X. Wang, X. Yangguang and L. Boran have built a temperature model for HRG and compensate the frequency based on the temperature [14,15,16,17]. X. Wang has studied a method to restrain the quadrature error [18]. For CVG, Innalab Inc., Waston Inc. have already designed a mature product, and have studied the error characteristics and performance [19,20]. P.W. Loveday, Y. Wu and D. Kristiansen have studied the temperature characteristics and error characteristics for CVG [21,22,23]. However, there is no error compensation method in the published literature.

This paper mainly studies the problem with the error model and compensation. A dynamic equation is firstly established, based on a study of the BVG working mechanism. This equation is used to evaluate the relationship between the angular rate output signal and the bell-shaped resonator characteristics, analyze the influence of the main error sources and set up an error model for the BVG. The error sources are classified using the error propagation properties, and a compensation method is presented based on the error model. Finally, the error model and compensation method are used to experimentally calibrate the BVG, including rough compensation, temperature and bias compensation, scale factor compensation and noise filter.

## 2. Working Concept of the Bell-Shaped Vibratory Gyro

**Figure 1:**
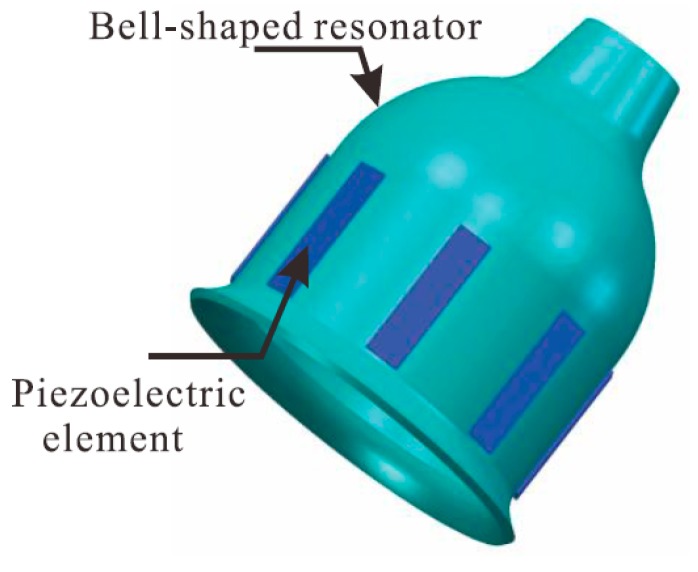
Structural diagram of Bell-shaped resonator.

The bell-shaped resonator is a core component of the BVG and looks like a millimeter-scaled Chinese traditional bell, such as the QianLong Bell or the YongLe Bell, shown in Figure 1. Eight piezoelectric elements are attached to the wall of the bell-shaped resonator, which excite the resonator and detect the signal to calculate the angular rate.

### 2.1. Working Principle

**Figure 2:**
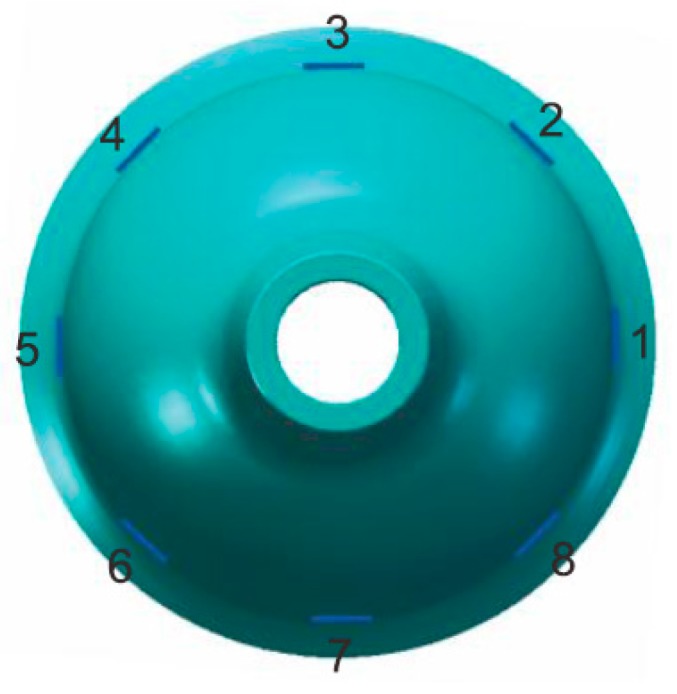
Schematic of mounted electrode.

**Figure 3:**
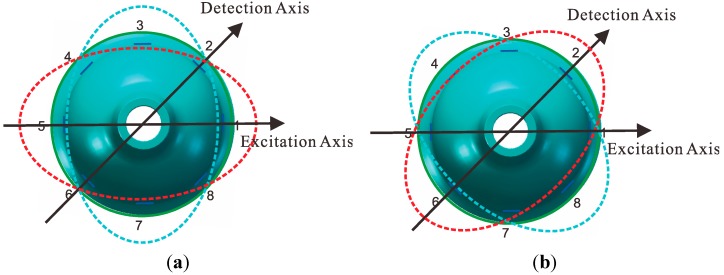
Schematic of the working principle (**a**) Primary mode (**b**) Secondary mode.

**Figure 4:**
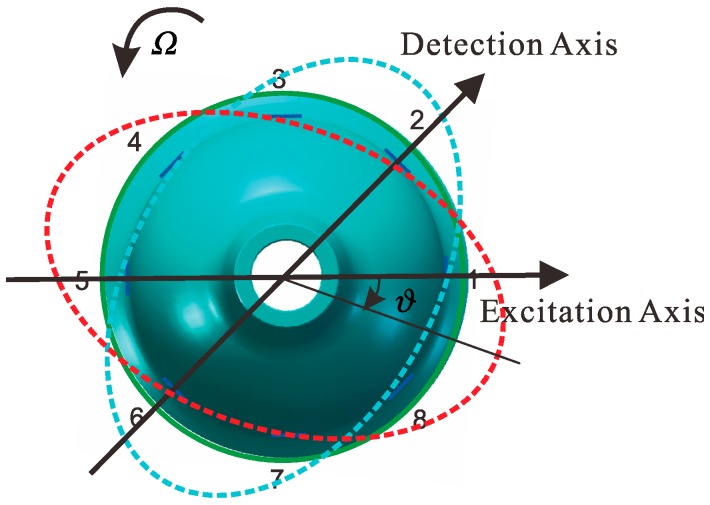
Schematic of the standing wave precession.

The eight piezoelectric elements are distributed evenly around the wall of bell-shaped resonator, as shown in Figure 2. Based on the inverse piezoelectric effect, the excitation electrodes excite the bell-shaped resonator and produce a working mode within the resonator, which is named the primary mode or the excitation mode, shown in Figure 3a. For the resonator, each working frequency has two modes, which are different by 45°. The other mode is named the secondary mode or detection mode, shown in Figure 3b. The two modes are coupled by the Coriolis force. The amplitude of the second mode is proportional to the input angular velocity and produces the standing wave precession. In Figure 4, when an angular velocity is applied counterclockwise to the axis of symmetry, the standing wave angle changes. The precession angle is −ϑ, which is proportional to the frame rotational angular velocity.

### 2.2. Excitation and Detection of Piezoelectricity

In practical applications, the stress wave propagates on the resonator, causing a standing wave. The piezo elements sensitize the stress wave and solve the angular velocity. In order to reduce the influence of the piezoelectric elements’ quality when the resonator is rotated, the elements should be set to be near the constrained boundary. However, in order to improve the excitation efficiency and stability, the piezoelectric should be glued on a flat surface such as a cylindrical structure.

**Figure 5:**
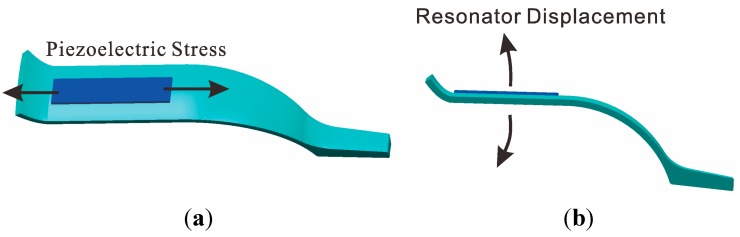
Schematic of piezoelectric principle.

The piezoelectric elements chosen were the PZT5A, which were polarized in the thickness direction. The first and fifth elements contract and expand when the alternating current signal is applied (in Figure 5a). When the top of the elements are restrained, the force of the contraction and expansion will be transferred to the bending force (in Figure 5b) and excite the shell vibration. The piezoelectric elements and resonator are attached together by conductive adhesives. A rigid connection can be made between these elements by controlling the painting procedure. One pole of elements connected to the resonator is the GND, and the remaining elements are the input or output signals. The piezoelectric element senses the vibratory signal based on the piezoelectric effect. Using the third and seventh element feedback signals and the first and fifth excitation elements, standing wave steady control is achieved. The control loop includes an amplitude control loop and a frequency control loop. It should be given particular emphasis to the fact that the work mode of the BVG is the force-rebalance mode [7,21]. The sensitized angle of precession from the second and sixth elements is used as the controller input to drive the fourth and eight elements to restrain the precession of the standing wave. The amplitude of the restrain variable is proportional to the angular velocity and is used as the output signal of the BVG [8].

## 3. Error Model of Bell-Shaped Vibratory Gyro

A bell-shaped resonator includes a hemispherical structure, a cylindrical structure and a hyperbolical structure. For this special multi-curved surface structure, the orthogonal curvilinear coordinate system is chosen to describe the bell-shaped resonator coordinates. The orthogonal curvilinear coordinate system uses the direction of the angle between the normal of the shell’s middle surface and the rotation axis, the rotation angle and the thickness to indicate a point of space. In [24], the author was given a detailed description of a bell-shaped resonator and deduced the curvature of a classic rotation shell. This coordinate system is used to research the characteristics of the bell-shaped resonator.

### 3.1. Dynamic Equation of Resonator’s Bottom Edge

**Figure 6:**
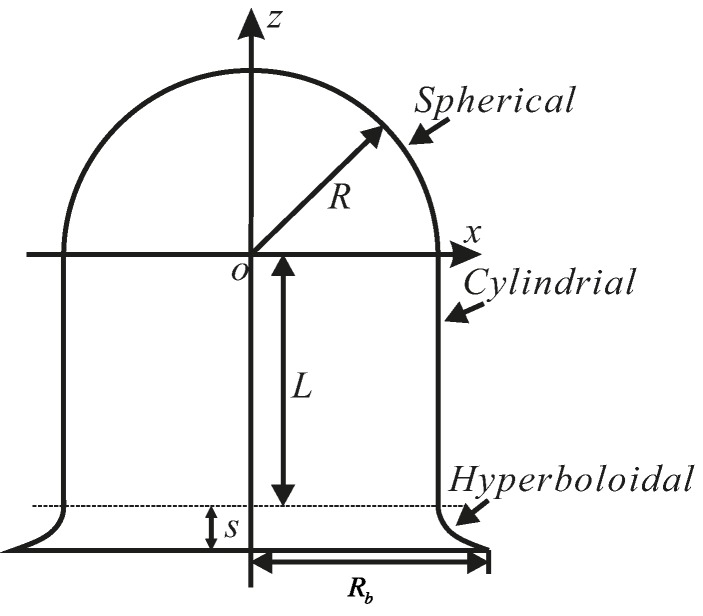
The middle surface coordinates of a bell-shaped resonator.

The middle surface coordinates of a bell-shaped resonator on an orthogonal curvilinear coordinate system is shown in Figure 6. The coordinate-origin is defined as the center of the hemispherical structure. The direction of the z axis is the rotation axis. The radius of the hemisphere is R. The height of the cylinder is *L* and the hyperbola is *S*. The radius of the bottom is $R_{b}$. The formula in Equation (1) can therefore be derived:

(1) $$\begin{array}{l} Hemispherical:\;x^{2}+z^{2}=R^{2};\;\left(0\leq z<R\right)\\ Cylindrical:\;x=R,z=t;\;\left(-L\leq z<0\right)\\ Hyperbolical:\;\frac{x^{2}}{R^{2}}-\frac{\left({R_{b}}^{2}-R^{2}\right){\left(z+L\right)}^{2}}{R^{2}S^{2}}=1;\;\left(-L-S\leq z<-L\right)\; \end{array}$$

**Figure 7:**
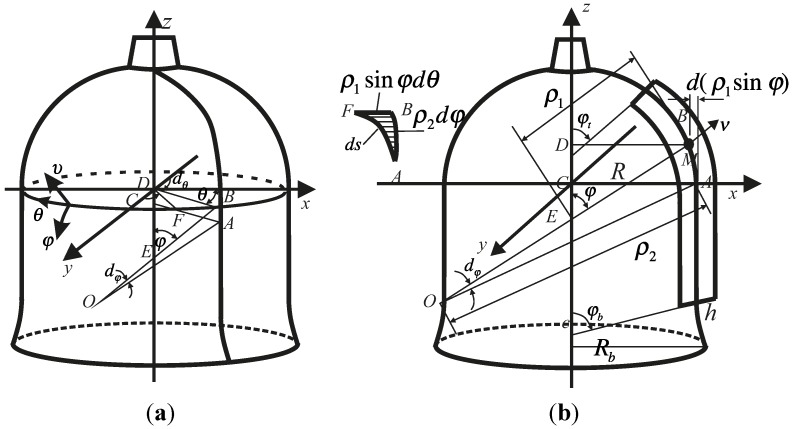
Schematic of bell-shaped resonator in coordinate system.

For the three-dimensional structure of the bell-shaped resonator, a description is shown in Figure 7 using the orthogonal curvilinear coordinate system (φ,υ,θ). The two principal radii of curvature are ${\text{ρ}}_{1}$ and ${\text{ρ}}_{2}$. φ is the angle between the normal of the point and the rotation axis. θ is the angle of the circumference. υ is the direction of thickness. ${\text{φ}}_{t}$ is the angle of the top and ${\text{φ}}_{b}$ is the angle of the bottom. h is the thickness of the resonator. The domain of definition of the resonator in orthogonal curvilinear coordinates is as given in Equation (2).

(2) $${\text{φ}}_{t}\leq \text{φ}\leq {\text{φ}}_{b},-\frac{h}{2}\leq \text{υ}\leq \frac{h}{2},\;0\leq \text{θ}<2\pi$$

The principal radii of curvature are as follows in Equation (3).

(3) $$\begin{array}{l} Hemispherical:\;{\text{ρ}}_{1}=R,{\text{ρ}}_{2}=R\\ Cylindrical:\;{\text{ρ}}_{1}=\infty ,{\text{ρ}}_{2}=R\\ Hyperbolical:\;{\text{ρ}}_{1}=\frac{-R^{4}S^{2}\sqrt{{R_{b}}^{2}-R^{2}}}{{\left[\left({R_{b}}^{2}-R^{2}\right)R^{2}\sin^{2}\text{φ}-R^{2}S^{2}\cos^{2}\text{φ}\right]}^{\frac{3}{2}}},\\ \;{\text{ρ}}_{2}=\frac{R^{2}\sqrt{{R_{b}}^{2}-R^{2}}}{{\left[\left({R_{b}}^{2}-R^{2}\right)R^{2}\sin^{2}\text{φ}-R^{2}S^{2}\cos^{2}\text{φ}\right]}^{\frac{1}{2}}} \end{array}$$

Therefore, many relationships between the coordinate system can be established, as follows

(4) $${\text{ρ}}_{z}={\text{ρ}}_{1}+\text{υ};\;r_{z}=\left({\text{ρ}}_{2}+\text{υ}\right)\sin\text{φ};\;\frac{d{\text{ρ}}_{z}}{dz}=1;\;\frac{d{\text{ρ}}_{z}}{d\text{φ}}=\frac{d{\text{ρ}}_{1}}{d\text{φ}}$$

The stress-strain relationship is as follows:

(5) $$\begin{array}{l} {\text{σ}}_{\text{φφ}}=\lambda \varepsilon +2G\varepsilon ,\;{\text{σ}}_{\text{υυ}}=\lambda \varepsilon +2G\varepsilon_{\text{υυ}},\;{\text{σ}}_{\text{θθ}}=\lambda \varepsilon +2G\varepsilon_{\text{θθ}}\\ {\text{σ}}_{\text{φυ}}=2G\varepsilon_{\text{φυ}},{\text{σ}}_{\text{φθ}}=2G\varepsilon_{\text{ϕθ}},{\text{σ}}_{\text{υθ}}=2G\varepsilon_{\text{υθ}} \end{array}$$

where σ are the stress components and the subscript is the axis, and ε are the strain components. G is the shear modulus and $G=\frac{E}{2\left(1+\gamma \right)}$. E is Young’s modulus. γ is Poisson’s ratio. ρ is the density of material. $\lambda =\frac{2G\gamma }{1-2\gamma }$.

The displacement components are as follows:

(6) $$\begin{array}{l} \varepsilon_{\text{φφ}}=\frac{1}{{\text{ρ}}_{z}}\left(\frac{\partial u_{\text{φ}}}{\partial \text{φ}}+u_{\text{υ}}\right);\varepsilon_{\text{υυ}}=\frac{\partial u_{\text{υ}}}{\partial \text{υ}};\varepsilon_{\text{θθ}}=\frac{1}{r_{z}}\left(\frac{\partial u_{\text{θ}}}{\partial \text{θ}}+u_{\text{φ}}\cos\text{φ}+u_{\text{υ}}\sin\text{φ}\right)\\ \varepsilon_{\text{υφ}}=\frac{1}{2}\left[\frac{\partial u_{\text{φ}}}{\partial \text{υ}}-\frac{1}{{\text{ρ}}_{z}}\left(u_{\text{φ}}-\frac{\partial u_{\text{υ}}}{\partial \text{φ}}\right)\right];\varepsilon_{\text{φθ}}=\frac{1}{2}\left[\frac{1}{r_{z}}\left(\frac{\partial u_{\text{φ}}}{\partial \text{θ}}-u_{\text{θ}}\cos\text{φ}\right)+\frac{\frac{\partial u_{\text{θ}}}{\partial \text{φ}}}{{\text{ρ}}_{z}}\right]\\ \varepsilon_{\text{υθ}}=\frac{1}{2}\left[\frac{1}{r_{z}}\left(\frac{\partial u_{\text{υ}}}{\partial \text{θ}}-u_{\text{θ}}\sin\text{φ}\right)+\frac{\partial u_{\text{θ}}}{\partial \text{υ}}\right];\varepsilon =\varepsilon_{\text{φφ}}+\varepsilon_{\text{υυ}}+\varepsilon_{\text{θθ}} \end{array}$$

where $u_{\text{φ}}$, $u_{\text{υ}}$ and $u_{\text{θ}}$ are the displacements in each of the three direction separately. In paper [25], the equation of motion in terms of the physical components is derived for the curvilinear (φ,υ,θ) coordinate system based on tensor analysis as follows:

(7) $$\begin{array}{c} \frac{\partial {\text{σ}}_{\text{φυ}}}{\partial \text{υ}}+\frac{1}{r_{z}}\left[\left({\text{σ}}_{\text{φφ}}-{\text{σ}}_{\text{θθ}}\right)\cos\text{φ}+\frac{\partial {\text{σ}}_{\text{φθ}}}{d\text{θ}}+{\text{σ}}_{\text{φθ}}\sin\text{φ}\right]+\frac{1}{{\text{ρ}}_{z}}\left(\frac{\partial {\text{σ}}_{\text{φφ}}}{\partial \text{φ}}+2{\text{σ}}_{\text{φυ}}\right)-\frac{2{\text{σ}}_{\text{φφ}}\frac{\partial {\text{ρ}}_{1}}{\partial \text{φ}}}{{{\text{ρ}}_{z}}^{2}}=-f_{\text{φ}}\\ \frac{\partial {\text{σ}}_{\text{υυ}}}{\partial \text{υ}}+\frac{1}{r_{z}}\left[\left({\text{σ}}_{\text{υυ}}-{\text{σ}}_{\text{θθ}}\right)\sin\text{φ}+\frac{\partial {\text{σ}}_{\text{υθ}}}{\partial \text{θ}}+{\text{σ}}_{\text{φυ}}\cos\text{φ}\right]+\frac{1}{{\text{ρ}}_{z}}\left(\frac{\partial {\text{σ}}_{\text{φυ}}}{\partial \text{φ}}+{\text{σ}}_{\text{υυ}}-{\text{σ}}_{\text{φφ}}\right)-\frac{{\text{σ}}_{\text{φυ}}\frac{\partial {\text{ρ}}_{1}}{\partial \text{φ}}}{{{\text{ρ}}_{z}}^{2}}=-f_{\text{υ}}\\ \frac{\partial {\text{σ}}_{\text{υθ}}}{\partial \text{υ}}+\frac{1}{r_{z}}\left(3{\text{σ}}_{\text{φθ}}\cos\text{φ}+2{\text{σ}}_{\text{υθ}}\sin\text{φ}+\frac{\partial {\text{σ}}_{\text{θθ}}}{\partial \text{θ}}\right)+\frac{1}{{\text{ρ}}_{z}}\left({\text{σ}}_{\text{υθ}}+\frac{\partial {\text{σ}}_{\text{φθ}}}{\partial \text{θ}}-\frac{{\text{σ}}_{\text{φθ}}}{r_{z}}\frac{\partial r_{z}}{\partial \text{φ}}\right)-\frac{{\text{σ}}_{\text{φθ}}\frac{\partial {\text{ρ}}_{1}}{\partial \text{φ}}}{{{\text{ρ}}_{z}}^{2}}=-f_{\text{θ}} \end{array}$$

When the BVG is in working mode, the bell-shaped resonator is influenced by the frame angular velocity. For a point P(φ,υ,θ), the motion expression of the vector is expressed as in Equation (8).

(8) $$\varphi =u_{\varphi }\hat{\varphi },\;\text{υ}=u_{\text{υ}}\hat{\text{υ}},\;\text{θ}\text{=}u_{\text{θ}}\hat{\text{θ}}$$

where φ, υ and θ are the vectors of the coordinate axis and $\hat{\text{φ}}$, $\hat{\text{υ}}$ and $\hat{\text{θ}}$ are the unit vectors of the coordinate axis. The motion vector ℜ of point *P* is as follows:

(9) $$ℜ=u_{\text{φ}}\hat{\text{φ}}+u_{\text{υ}}\hat{\text{υ}}+u_{\text{θ}}\hat{\text{θ}}$$

The angular velocity Ω along the axis of symmetry in the inertial space is:

(10) $$\Omega =\Omega \left(\cos\text{φ}\hat{\text{υ}}-\sin\text{φ}\hat{\text{φ}}\right)=\Omega \cos\text{φ}\hat{\text{υ}}-\Omega \sin\text{φ}\hat{\text{φ}}$$

According to the Coriolis Theorem, the absolute acceleration of point *P* in motion relative to inertial space can be expressed as:

(11) $$a=a_{0}+2\Omega \times \dot{ℜ}+\ddot{ℜ}+\left[\dot{\Omega }\times ℜ\right]+\left[\Omega \times \left[\Omega \times ℜ\right]\right]$$

where $a_{0}$ is the absolute acceleration of the bell-shaped resonator. $\ddot{ℜ}$ is the acceleration of *P* for the resonator. $\dot{ℜ}$ is the velocity of *P* for the resonator. $\dot{\Omega }$ is the angular acceleration of the resonator.

According to the Equation (11), the absolute acceleration of *P* is as follows:

(12) $$\begin{array}{l} a_{\text{φ}}={\ddot{u}}_{\text{φ}}+\dot{\Omega }u_{\text{θ}}\cos\text{φ}+2\Omega {\dot{u}}_{\text{θ}}\cos\text{φ}-\Omega^{2}u_{\text{φ}}\cos^{2}\text{φ}-\Omega^{2}u_{\text{υ}}\cos\text{φ}\sin\text{φ}\\ a_{\text{υ}}={\ddot{u}}_{\text{υ}}+\dot{\Omega }u_{\text{θ}}\sin\text{φ}-\Omega^{2}u_{\text{υ}}+2\Omega {\dot{u}}_{\text{θ}}\sin\text{φ}+\Omega^{2}u_{\text{υ}}\cos^{2}\text{φ}-\Omega^{2}u_{\text{φ}}\sin\text{φ}\cos\text{φ}\\ a_{\text{θ}}={\ddot{u}}_{\text{θ}}-\Omega^{2}u_{\text{θ}}-\dot{\Omega }u_{\text{φ}}\cos\text{φ}-\dot{\Omega }u_{\text{υ}}\sin\text{φ}-2\Omega {\dot{u}}_{\text{φ}}\cos\text{φ}-2\Omega {\dot{u}}_{\text{υ}}\sin\text{φ} \end{array}$$

where, $a_{\text{φ}}$, $a_{\text{υ}}$ and $a_{\text{θ}}$ are the absolute accelerations in the coordinate direction.

The natural frequency of BVG is designed to be below 10 kHz. The range is below 5000 deg/s. For these low velocity fields, the angular acceleration of the resonator and the angular squared term can be ignored. The expression of acceleration at the free edge of the resonator is then as follows:

(13) $$a_{\text{φ}}={\ddot{u}}_{\text{φ}}+2\Omega {\dot{u}}_{\text{θ}}\cos\text{φ};\;a_{\text{υ}}={\ddot{u}}_{\text{υ}}+2\Omega {\dot{u}}_{\text{θ}}\sin\text{φ};\;a_{\text{θ}}={\ddot{u}}_{\text{θ}}-2\Omega {\dot{u}}_{\text{υ}}\cos\text{φ}$$

According to Newton’s Second Law, the inertial force of *P* is influenced by angular velocity as follows:

(14) $$\begin{array}{l} f_{\text{φ}}=-\text{ρ}ha_{\text{φ}}=-\text{ρ}h\left({\ddot{u}}_{\text{φ}}+2\Omega {\dot{u}}_{\text{θ}}\cos\text{φ}\right)\\ f_{\text{υ}}=-\text{ρ}ha_{\text{υ}}=-\text{ρ}h\left({\ddot{u}}_{\text{υ}}+2\Omega {\dot{u}}_{\text{θ}}\sin\text{φ}\right)\\ f_{\text{θ}}=-\text{ρ}ha_{\text{θ}}=-\text{ρ}h\left({\ddot{u}}_{\text{θ}}-2\Omega {\dot{u}}_{\text{υ}}\cos\text{φ}-2\Omega {\dot{u}}_{\text{υ}}\sin\text{φ}\right) \end{array}$$

Substituting Equation (14) into Equation (7) derives the motion equation of the bell-shaped resonator’s edge when the angular velocity Ω is along the rotation axis:

(15) $$\begin{array}{c} \frac{\partial {\text{σ}}_{\text{φυ}}}{\partial \text{υ}}+\frac{1}{r_{z}}\left[\left({\text{σ}}_{\text{φφ}}-{\text{σ}}_{\text{θθ}}\right)\cos\text{φ}+\frac{\partial {\text{σ}}_{\text{φθ}}}{\partial \text{θ}}+{\text{σ}}_{\text{φθ}}\sin\text{φ}\right]\\ +\frac{1}{{\text{ρ}}_{z}}\left(\frac{\partial {\text{σ}}_{\text{φφ}}}{\partial \text{φ}}+2{\text{σ}}_{\text{φυ}}\right)-\frac{2{\text{σ}}_{\text{φφ}}\frac{\partial {\text{ρ}}_{1}}{\partial \text{φ}}}{{{\text{ρ}}_{z}}^{2}}=\text{ρ}h\left({\ddot{u}}_{\text{φ}}+2\Omega {\dot{u}}_{\text{θ}}\cos\text{φ}\right)\\ \frac{\partial {\text{σ}}_{\text{υυ}}}{\partial \text{υ}}+\frac{1}{r_{z}}\left[\left({\text{σ}}_{\text{υυ}}-{\text{σ}}_{\text{θθ}}\right)\sin\text{φ}+\frac{\partial {\text{σ}}_{\text{υθ}}}{\partial \text{θ}}+{\text{σ}}_{\text{φυ}}\cos\text{φ}\right]\\ +\frac{1}{{\text{ρ}}_{z}}\left(\frac{\partial {\text{σ}}_{\text{φυ}}}{\partial \text{φ}}+{\text{σ}}_{\text{υυ}}-{\text{σ}}_{\text{φφ}}\right)-\frac{{\text{σ}}_{\text{φυ}}\frac{\partial {\text{ρ}}_{1}}{\partial \text{φ}}}{{{\text{ρ}}_{z}}^{2}}=\text{ρ}h\left({\ddot{u}}_{\text{υ}}+2\Omega {\dot{u}}_{\text{θ}}\sin\text{φ}\right)\\ \frac{\partial {\text{σ}}_{\text{υθ}}}{\partial \text{υ}}+\frac{1}{r_{z}}\left(3{\text{σ}}_{\text{φθ}}\cos\text{φ}+2{\text{σ}}_{\text{υθ}}\sin\text{φ}+\frac{\partial {\text{σ}}_{\text{θθ}}}{\partial \text{θ}}\right)\\ +\frac{1}{{\text{ρ}}_{z}}\left({\text{σ}}_{\text{υθ}}+\frac{\partial {\text{σ}}_{\text{φθ}}}{\partial \text{θ}}-\frac{{\text{σ}}_{\text{φθ}}}{r_{z}}\frac{\partial r_{z}}{\partial \text{φ}}\right)-\frac{{\text{σ}}_{\text{φθ}}\frac{\partial {\text{ρ}}_{1}}{\partial \text{φ}}}{{{\text{ρ}}_{z}}^{2}}=\text{ρ}h\left({\ddot{u}}_{\text{θ}}-2\Omega {\dot{u}}_{\text{υ}}\cos\text{φ}-2\Omega {\dot{u}}_{\text{υ}}\sin\text{φ}\right) \end{array}$$

Then, substituting Equations (5) and (6) into Equation (15) derives the model of the bell-shaped resonator. These equations are also named Navier’s displacement equations of motion. For analysis of the model, it is assumed that the bell-shaped resonator is perfect. The material parameters have a constant value and are irrelevant to the circle angle. The model is derived using the Maple program which is too complex to describe in detail here.

To solve the model, the displacement vector of an arbitrary point in the bell-shaped resonator is developed according to the second order normal vibration mode, which is not stretched [4]:

(16) $$\left[\begin{array}{c} u_{\text{φ}}\left(\text{φ},\text{θ},t\right)\\ u_{\text{υ}}\left(\text{φ},\text{θ},t\right)\\ u_{\text{θ}}\left(\text{φ},\text{θ},t\right) \end{array}\right]=\left[\begin{array}{c} U\left(\text{φ}\right)\cos2\text{θ}\\ V\left(\text{φ}\right)\sin2\text{θ}\\ W\left(\text{φ}\right)\cos2\text{θ} \end{array}\right]p\left(t\right)+\left[\begin{array}{c} U\left(\text{φ}\right)\sin2\text{θ}\\ -V\left(\text{φ}\right)\cos2\text{θ}\\ W\left(\text{φ}\right)\sin2\text{θ} \end{array}\right]q\left(t\right)$$

where U(φ), V(φ), W(φ) are the Rayleigh functions for the bell-shaped resonator’s second order intrinsic vibration mode on the three axes. p(t) and q(t) are the displacements of the vibratory rigid axis.

Based on the hypothesis that the shell’s middle surface is not stretched, the sheer displacement of the bend shell is equal to zero, and is as follows [4]:

(17) $$\varepsilon_{\text{φφ}}=\varepsilon_{\text{θθ}}=\varepsilon_{\text{φθ}}=0$$

Substituting Equation (6) into Equation (17) gives the following formula:

(18) $$\left\{ \begin{array}{l} \frac{\partial u_{\text{φ}}}{\partial \text{φ}}+u_{\text{υ}}=0\\ \frac{\partial u_{\text{θ}}}{\partial \text{θ}}+u_{\text{φ}}\cos\text{φ}+u_{\text{υ}}\sin\text{φ}=0\\ \frac{1}{r_{z}}\left(\frac{\partial u_{\text{φ}}}{\partial \text{θ}}-u_{\text{θ}}\cos\text{φ}\right)+\frac{\frac{\partial u_{\text{θ}}}{\partial \text{φ}}}{{\text{ρ}}_{z}}=0 \end{array}\right.$$

Substituting Equations (16) into (18), the Rayleigh function of the resonator’s second order normal vibration mode is derived using the separation of variables method [8].

(19) $$\begin{array}{l} U\left(\text{φ}\right)=-A_{k}\cos\left(\frac{5\pi \text{φ}}{{\text{φ}}_{b}}\right)\sin^{3}\text{φ}\\ V\left(\text{φ}\right)=-A_{k}\cos\text{φ}\sin^{3}\text{φ}\\ W\left(\text{φ}\right)=A_{k}3\cos\text{φ}\sin^{2}\text{φ} \end{array}$$

where $A_{k}$ is the amplitude function of the bell-shaped resonator. For convenient calculation, $A_{k}$ is defined to be constant with value 1.

Substituting Equation (5), Equation (6) and Equation (19) into Equation (15), the dynamic equation of the bell-shaped resonator’s second order normal vibration mode using Bubnov-Galyorkin method is:

(20) $$\left\{ \begin{array}{l} m_{0}\ddot{p}\left(t\right)-2\Omega b\dot{q}\left(t\right)+c_{0}p\left(t\right)+c_{1}q\left(t\right)=0\\ m_{0}\ddot{q}\left(t\right)+2\Omega b\dot{p}\left(t\right)+c_{0}q\left(t\right)+c_{1}p\left(t\right)=0 \end{array}\right.$$

where $m_{0}=-\text{ρ}h\pi W\left(\text{φ}\right)$, b=ρhπV(φ)(cosφ+sinφ),

$$\begin{array}{c} \begin{array}{c} c_{0}=\pi \{-\frac{G\left(-W\sin\text{φ}+2V\right)}{{\left({\text{ρ}}_{2}+\text{υ}\right)}^{2}\sin\text{φ}}+\frac{1}{\left({\text{ρ}}_{2}+\text{υ}\right)\sin\text{φ}}[\frac{2G\left(-W\sin\text{φ}+2V\right)}{{\text{ρ}}_{2}+\text{υ}}+\frac{2G\left(2V\sin\text{φ}-4W\right)}{\left({\text{ρ}}_{2}+\text{υ}\right)\sin\text{φ}}\\ +6G(-\frac{1}{2}\frac{W\cos\text{φ}}{\left({\text{ρ}}_{2}+\text{υ}\right)\sin\text{φ}}+\frac{1}{2}\frac{W^{\prime }}{{\text{ρ}}_{1}+\text{υ}})\cos\text{φ}+(\frac{2V}{{\text{ρ}}_{1}+\text{υ}}+\frac{2\sin\text{φ}V-4W}{\left({\text{ρ}}_{2}+\text{υ}\right)\sin\text{φ}})\lambda ]\\ +\frac{1}{{\text{ρ}}_{1}+\text{υ}}[\frac{G\left(2V-W\sin\text{φ}-4U+\frac{W\cos\text{φ}}{\left({\text{ρ}}_{2}+\text{υ}\right)\sin\text{φ}}-\frac{W^{\prime }}{{\text{ρ}}_{1}+\text{υ}}\right)\left(\frac{d{\text{ρ}}_{2}}{d\text{φ}}\sin\text{φ}+\left({\text{ρ}}_{2}+\text{υ}\right)\cos\text{φ}\right)}{\left({\text{ρ}}_{2}+\text{υ}\right)\sin\text{φ}}],\\ -\frac{G\left(-\frac{W\cos\text{φ}}{\left({\text{ρ}}_{2}+\text{υ}\right)\sin\text{φ}}+\frac{W^{\prime }}{{\text{ρ}}_{1}+\text{υ}}\right)\left(\frac{d{\text{ρ}}_{1}}{d\text{φ}}\right)}{{\left({\text{ρ}}_{1}+\text{υ}\right)}^{2}}\} \end{array}\\ \begin{array}{c} c_{1}=\pi [\frac{-\frac{10GU\cos\text{φ}}{\left({\text{ρ}}_{2}+\text{υ}\right)\sin\text{φ}}+2\lambda \left(-\frac{U^{\prime }}{{\text{ρ}}_{1}+\text{υ}}-\frac{U\cos\text{φ}}{\left({\text{ρ}}_{2}+\text{υ}\right)\sin\text{φ}}\right)}{\left({\text{ρ}}_{2}+\text{υ}\right)\sin\text{φ}}+\frac{2GU\frac{d{\text{ρ}}_{1}}{d\text{φ}}}{\left({\text{ρ}}_{2}+\text{υ}\right){\left({\text{ρ}}_{1}+\text{υ}\right)}^{2}\sin\text{φ}}\\ +\frac{2G\left(\frac{W\cos\text{φ}}{\left({\text{ρ}}_{2}+\text{υ}\right)\sin\text{φ}}-\frac{W^{\prime }}{{\text{ρ}}_{1}+\text{υ}}\right)+\frac{2GU\left(\frac{d{\text{ρ}}_{2}}{d\text{φ}}\sin\text{φ}+\left({\text{ρ}}_{2}+\text{υ}\right)\cos\text{φ}\right)}{{\left({\text{ρ}}_{2}+\text{υ}\right)}^{2}\sin^{2}\text{φ}}}{{\text{ρ}}_{1}+\text{υ}}] \end{array}\\ U^{\prime }=\frac{dU}{d\text{φ}},\;V^{\prime }=\frac{dV}{d\text{φ}},\;W^{\prime }=\frac{dW}{d\text{φ}}. \end{array}$$

The characteristics of the bell-shaped resonator can be derived from the precession factor and natural frequency using Equation (20). It is demonstrated that the BVG is a Coriolis vibratory gyroscope which has the advantage of vibratory gyro. The motion of the bell-shaped resonator’s edge is shown to be equivalent to a two-dimensional spring. p(t) is equivalent to the axis between the 0° piezoelectric electrode and the 180° electrode. q(t) is equivalent to the axis between the 45° piezoelectric electrode to the 225° electrode. Based on the Equation (20), the precession factor can be obtained as follows:

(21) $$k=\frac{b}{2m_{0}}$$

The natural frequency is as follows:

(22) $${\text{ω}}_{n}=\sqrt{\frac{c_{0}}{m_{0}}}$$

### 3.2. Angular Velocity Measurement

**Figure 8:**
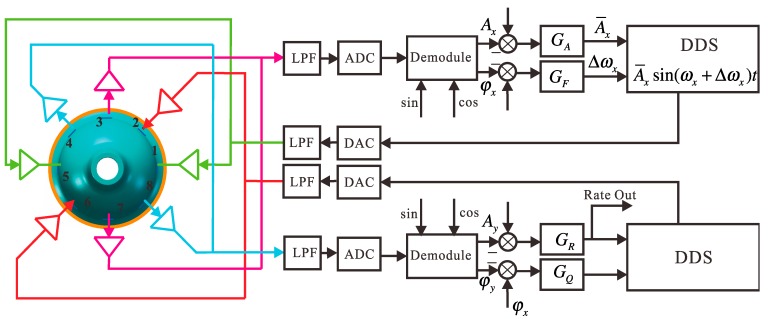
The circuit system signal flow.

Electrodes 1 and 5 in the *p*(*t*) shafting are the driving electrodes. A sinusoidal signal at the natural frequency of the bell-shaped resonator causes the resonator to vibrate. The driving signal is generated directly using DSP with the Direct Digital Synthesis (DDS) algorithm and is applied on Electrodes 1 and 5 with the DAC. DDS also supplies the exact modulating signal for calculating the amplitude and phase. It calculates the resonator’s vibration by detection at Electrodes 3 and 7. The bell-shaped resonator can be made to generate resonance by dynamically adjusting DDS through design of the amplitude loop controller *G_A_*, and frequency loop controller *G_F_*. Electrodes 4 and 8 are detected on the *q*(*t*) axis. By analyzing the standing wave’s procession, designing the rate loop controller *G_R_*, and quadrature loop controller *G_Q_*, and dynamically adjusting the damping torque on Electrodes 2 and 6, the gyro can be made to work in force rebalance mode, leaving the mode shape unchanged. At the same time, the output of the controller in the rate control loop is proportional to the input angular rate. The whole signal flow is shown in Figure 8.

For the circuit system’s hardware application, the control loops and DDS signals are generated from DSP. The signal collection is done by DAC. The analog conditioning circuit is used for detection and driving of the piezoelectric electrodes. In the real circuit system, we use the STM32F405 as the DSP chip, DSP to supply ADC and AD5328 as the DAC chip. The core component of the analog conditioning circuit is OPA2227 9.

BVG works in forced vibration. The main vibration force is applied by exciting the piezoelectric element. If the bell-shaped resonator is in a steady state as described by Equation (20), a force should be applied to the right of equation as shown in Equation (23):

(23) $$\left\{ \begin{array}{l} m_{0}\ddot{p}\left(t\right)-2\Omega b\dot{q}\left(t\right)+c_{0}p\left(t\right)+c_{1}q\left(t\right)=f_{p}\\ m_{0}\ddot{q}\left(t\right)+2\Omega b\dot{p}\left(t\right)+c_{0}q\left(t\right)+c_{1}p\left(t\right)=f_{q} \end{array}\right.$$

where $f_{p}=A_{p}\sin\left(2\pi {\text{ω}}_{p}t+{\text{ϕ}}_{p}\right)$ is the force applied along axis *p*, $A_{p}$ is the amplitude of $f_{p}$, ${\text{ω}}_{p}$ is the frequency of axis *p*, ${\text{ϕ}}_{p}$ is the phase of $f_{p}$. $f_{q}=A_{q}\sin\left(2\pi {\text{ω}}_{q}t+{\text{ϕ}}_{q}\right)$ is the damp force applied along axis *q*, $A_{q}$ is the amplitude of $f_{q}$, ${\text{ω}}_{q}$ is the frequency of axis *q*, ${\text{ϕ}}_{q}$ is the phase of $f_{q}$.

For this vibratory gyro, the excitation force applied on axis *p* is the key factor to keep the resonator in a resonant state. Ideally, $f_{p}$ should remain unchanged. It is assumed that the natural frequency is unchanged. $f_{q}$ will vary based on the angular velocity Ω, and its amplitude, frequency and phase depends on the working mode of BVG. The angular velocity Ω is as shown in Equation (24):

(24) $$\begin{array}{c} \Omega =\frac{f_{q}}{4k\dot{p}\left(t\right)}-\frac{\ddot{q}\left(t\right)+{\text{ω}}_{n}^{2}\dot{q}\left(t\right)+\frac{c_{1}}{m_{0}}p\left(t\right)}{4k\dot{p}\left(t\right)}\\ =\frac{f_{p}}{4k\dot{q}\left(t\right)}-\frac{\ddot{p}\left(t\right)+{\text{ω}}_{n}^{2}\dot{p}\left(t\right)+\frac{c_{1}}{m_{0}}q\left(t\right)}{4k\dot{q}\left(t\right)} \end{array}$$

where the vibration of axis *p* keeps the resonator in steady state depending on excitation $f_{p}$. The angular velocity is solved based on the displacement of axis *q*. In the open loop mode, the angular velocity is as shown in Equation (25). In the force rebalance mode, the angular velocity is as shown in Equation (26)

(25) $$\Omega =-\frac{\ddot{q}\left(t\right)+{\text{ω}}_{n}^{2}\dot{q}\left(t\right)+\frac{c_{1}}{m_{0}}p\left(t\right)}{4k\dot{p}\left(t\right)}$$

(26) $$\Omega =\frac{f_{q}}{4k\dot{p}\left(t\right)}-\frac{\ddot{q}\left(t\right)+{\text{ω}}_{n}^{2}\dot{q}\left(t\right)+\frac{c_{1}}{m_{0}}p\left(t\right)}{4k\dot{p}\left(t\right)}$$

For the BVG, the force balance mode is chosen, which can improve the accuracy, restrain precession and improve bandwidth. The displacement of p(t) and q(t) is solved by the detection piezoelectric element [9].

**Figure 9:**
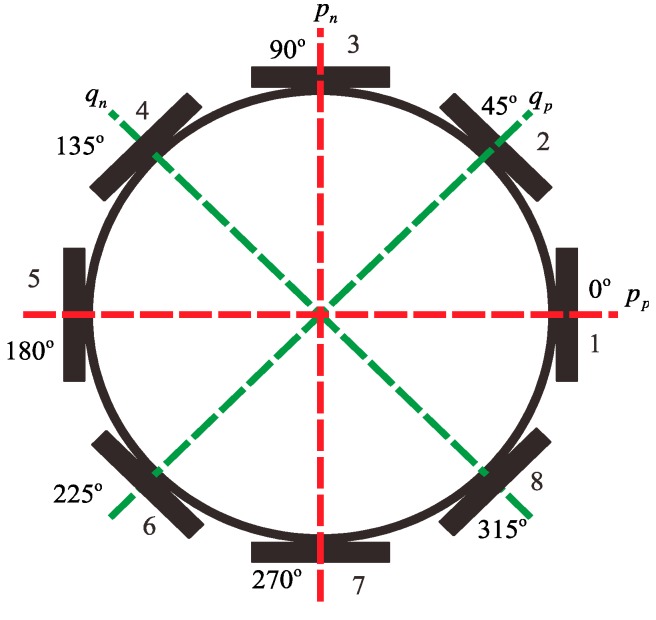
The piezoelectric element.

The piezoelectric elements are attached to the wall of the bell-shaped resonator as shown in Figure 9. The piezoelectric elements 3 and 7 measure the displacement of axis *p*, and the piezoelectric elements 4 and 8 measure the displacement of axis *q*. Based on the piezoelectric effect, the charge of piezoelectric 3, 7, 4 and 8 is

(27) $$F_{i}\left(t\right)=\left({\text{σ}}_{\nu \nu }+{\text{σ}}_{\text{φφ}}\right)d_{31}S_{i},\;i=3,4,7,8$$

where, $F_{i}\left(t\right)$ is the charge of the piezoelectric element (C), and $d_{31}$ is the piezoelectric constant of the piezoelectric element (C/N). $S_{i}$ is the area of the piezoelectric element in m^2^. Using the C-V converter circuit, the charge is converted to voltage which is collected by the ADC converter and provided to the DSP. For the digital process, the relationship is as follows:

(28) $$\left\{ \begin{array}{l} p\left(t\right)=\varsigma_{p}\left[F_{3}\left(t\right)+F_{7}\left(t\right)\right]\\ q\left(t\right)=\varsigma_{q}\left[F_{4}\left(t\right)+F_{8}\left(t\right)\right] \end{array}\right.$$

where, $\varsigma_{p}$ is the coefficient of displacement between the synthesized voltage of the piezoelectric elements 3 and 7 and $\varsigma_{q}$ is the coefficient of displacement between the synthesized voltage of piezoelectric elements 4 and 8.

### 3.3. Error Model

Based on the analysis results, the angular velocity of BVG can be calculated by the following expression:

(29) $$\Omega =\frac{A_{q}\sin\left({\text{ω}}_{q}t+{\text{ϕ}}_{q}\right)}{4k\varsigma_{p}\left[{\dot{F}}_{3}\left(t\right)+{\dot{F}}_{7}\left(t\right)\right]}-\frac{\varsigma_{q}\left[{\ddot{F}}_{4}\left(t\right)+{\ddot{F}}_{8}\left(t\right)\right]+{\text{ω}}_{n}^{2}\varsigma_{q}\left[{\dot{F}}_{4}\left(t\right)+{\dot{F}}_{8}\left(t\right)\right]+\frac{c_{1}}{m_{0}}\varsigma_{p}\left[F_{3}\left(t\right)+F_{7}\left(t\right)\right]}{4k\varsigma_{p}\left[{\dot{F}}_{3}\left(t\right)+{\dot{F}}_{7}\left(t\right)\right]}$$

During the implementation process, the loop controller selects the damp force $A_{q}$, frequency ${\text{ω}}_{q}$ and phase ${\text{ϕ}}_{q}$. The output angular velocity is expressed as

(30) $$\Omega =ΚA_{q}$$

where K is the coefficient of the controller, which is applied to the motion of the axes and controller algorithm.

Based on (29), the error sources of BVG include:

(1) ${\text{ω}}_{p}$, ${\text{ω}}_{q}$ and ${\text{ω}}_{n}$ which are all different;

The difference between ${\text{ω}}_{p}$ and ${\text{ω}}_{q}$, is known as the frequency split. Many restraining methods are given in other studies [11,12,23].

(2) The frequency of the resonator includes ${\text{ω}}_{p}$, ${\text{ω}}_{q}$ and ${\text{ω}}_{n}$, which are all influenced by temperature;

(3) The circuit devices are influenced by temperature;

(4) The precession factor is changed in different environments;

(5) The drift of the control loop algorithm;

(6) Noise.

Error Sources (2), (3) and (4) are difficult to compensate. These can be transferred to the gyro index which includes zero bias and a scale factor. Therefore, the error model can be described as:

(31) $$\Omega =SF\left(\Omega ,T\right)\cdot A_{q}\left(\xi \right)-N_{u}\left(T\right)+v\left(t\right)$$

where, SF(Ω,T) is the scale factor of the BVG which is determined by the angular velocity and temperature of the bell-shaped resonator. $A_{q}\left(\xi \right)$ is the output of the controller. $N_{u}\left(T\right)$ is the bias. v(t) is noise.

## 4. Compensation Principle

Based on Equation (31), the error sources can be classified into three types: bias compensation, scale factor compensation and noise filter. The error model can be described based on Equation (31) as:

(32) $$\Omega =SF_{3}(T)\cdot \{SF_{2}(\Omega )\cdot [SF_{1}\cdot (A_{q}-N_{u1})-N_{u}(T)]\}+v\left(t\right)$$

where $SF_{1}$ is the rough scale factor. $N_{u1}$ is the rough zero null. $N_{u}\left(T\right)$ is the bias due to temperature compensation. $SF_{2}\left(\Omega \right)$ is the scale factor due to angular velocity. $SF_{3}\left(T\right)$ is scale factor due to temperature. The compensation steps can be described as:

(1) Rough compensation: calculate $SF_{1}$ and $N_{u1}$.

(2) Bias compensation: calculate $N_{u}\left(T\right)$.

(3) Scale factor: calculate $SF_{2}\left(\Omega \right)$ and $SF_{3}\left(T\right)$.

(4) Noise: v(t).

### 4.1. Rough Compensation

**Figure 10:**
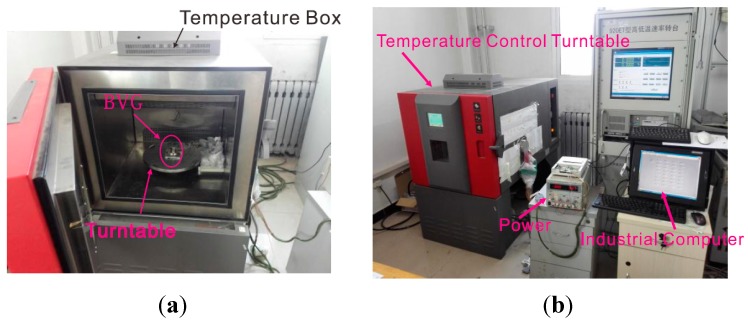
Photo of the experiment.

**Figure 11:**
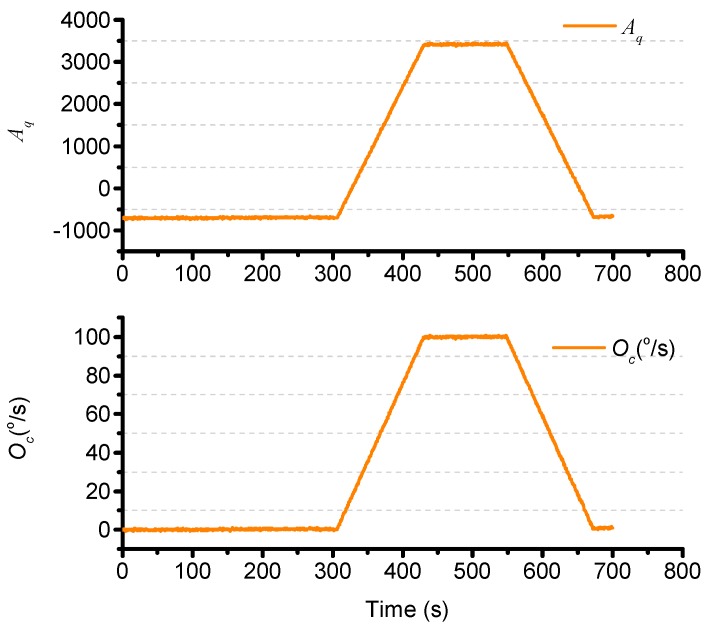
Rough compensation curve.

Firstly, BVG is fixed on the temperature control turntable to compensate for error, as shown in Figure 10. The output of BVG is $A_{q}$ and the output data is stored using an industrial computer. The update time of BVG is 100ms. The turntable remains stationary for five minutes, and then maintains a speed of +100°/s. The test curve is as shown in Figure 11.

As we can see in Figure 11, the rough null is −703.3858. The rough scale factor is 0.00242. The relationship between the output and angular velocity is then:

(33) $$\Omega_{C}=SF_{1}\cdot \left(A_{q}-N_{u1}\right)=\text{0.0242}\cdot \left(A_{q}+703.3858\right)$$

### 4.2. Bias Compensation

**Figure 12:**
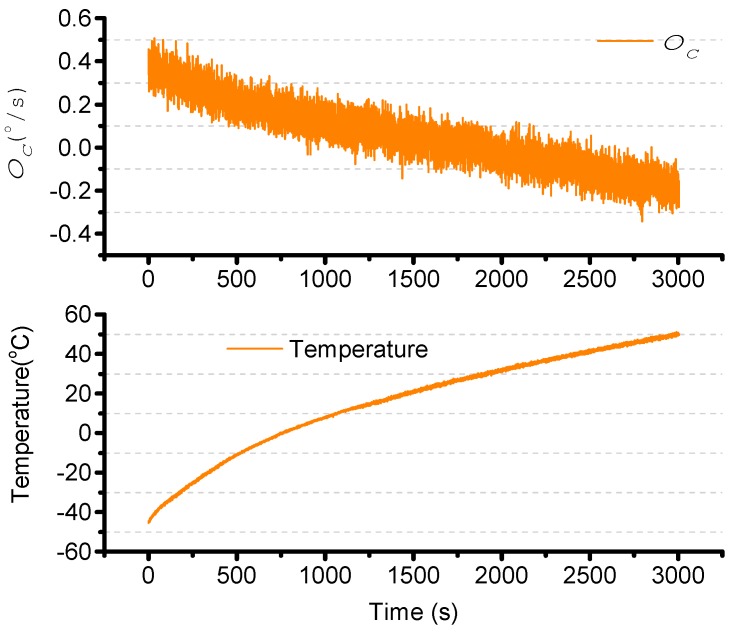
$\Omega_{C}$ in temperature changes.

The turntable is kept stationary and the temperature control box is maintained at −45 °C for 2 h. The temperature control box is then increased from −45 °C to +55 °C. $\Omega_{C}$ is stored, and is as shown in Figure 12. The bias instability is 20.5°/h.

**Figure 13:**
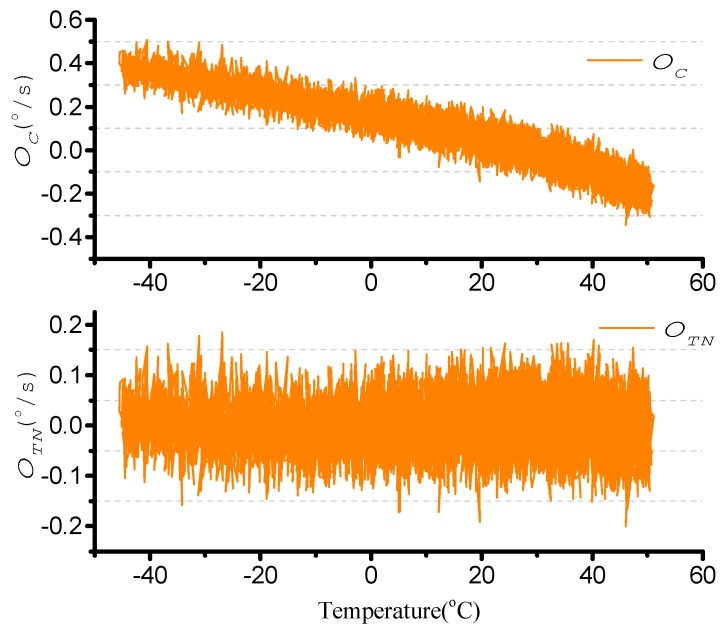
Temperature compensation.

The second-order least square compensation bias over work temperature is as expressed in Equation (34). The result is as shown in Figure 13. The standard deviation is from 0.1624°/s to 0.0619°/s.

(34) $$\begin{array}{cc} \Omega_{TN} & =\Omega_{C}-\left(a_{1}\cdot T^{2}+a_{2}\cdot T+a_{3}\right)\\ & =\Omega_{C}-\left(-\text{2.52e}-\text{05}\cdot T^{2}-0.0056\cdot T+0.1673\right) \end{array}$$

### 4.3. Scale Factor Compensation

**Figure 14:**
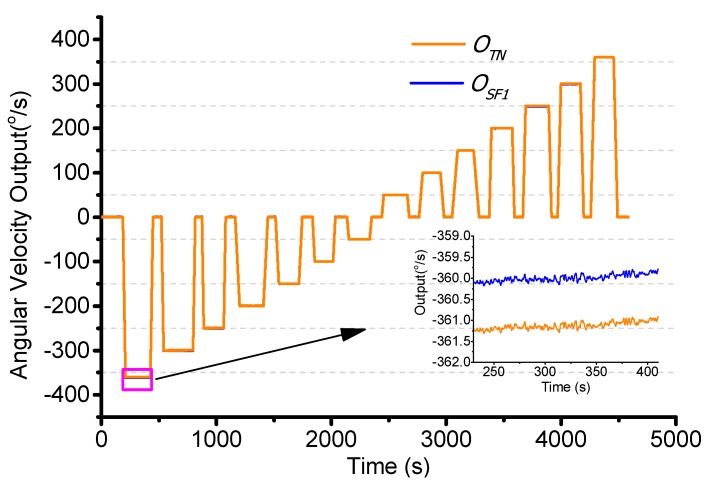
Scale factor changes with angular velocity.

**Figure 15:**
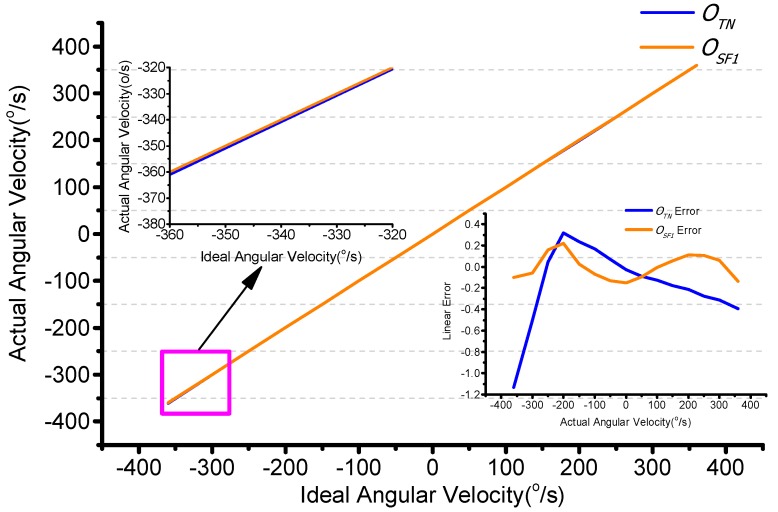
Scale factor compensation by angular velocity.

The scale factor error includes the angular velocity and temperature. Initially, the turntable is set to work from −360°/s to +360°/s at a constant temperature of 25 °C. The test result is shown in Figure 14. When the input angular velocity is −360°/s, the test is −361.4°/s. This shows that the scale factor changes with the input angular velocity. The linearity is 0.2% as shown in Figure 15.

The second order least square compensation bias over range is as expressed in Equation (35). The linearity error is from 0.2% to 0.03% as shown in Figure 15.

(35) $$\begin{array}{cc} \Omega_{SF1} & ={a_{1}}^{\prime }\cdot {\Omega_{TN}}^{2}+{a_{2}}^{\prime }\cdot \Omega_{TN}+{a_{3}}^{\prime }\\ & =\text{5.89e-06}\cdot {\Omega_{TN}}^{2}+\text{0.99}\cdot \Omega_{TN}\text{-0.12} \end{array}$$

**Figure 16:**
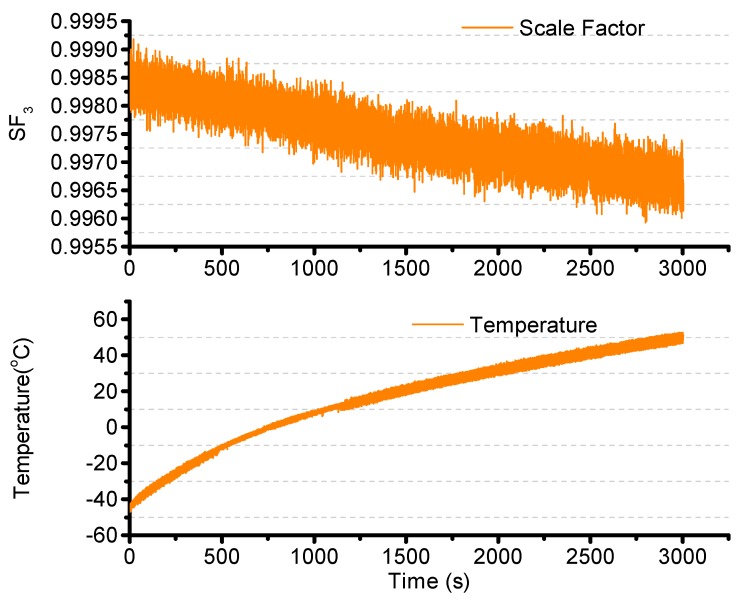
Scale factor changes with temperature.

The temperature error of the scale factor is then studied. The turntable is set to operate at 300°/s, and the temperature is increased from −45 °C to +55 °C. The result is shown in Figure 16.

**Figure 17:**
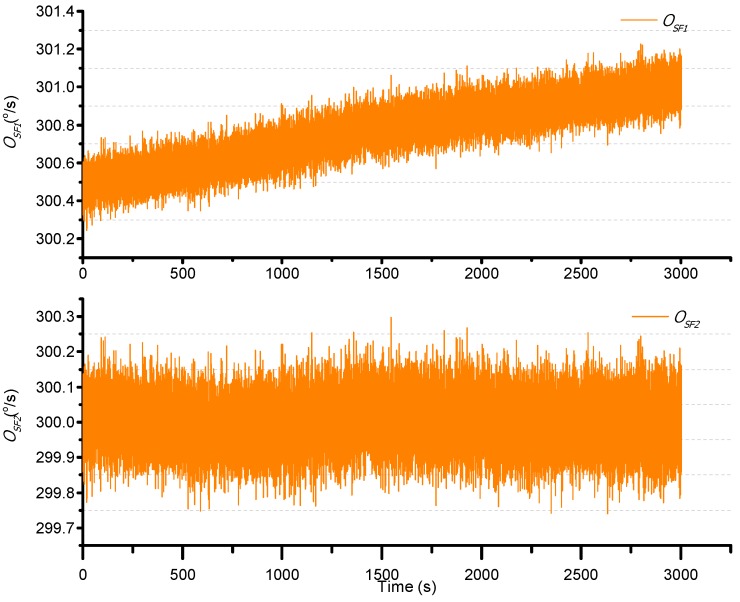
Temperature compensation.

The second order least square compensation bias over temperature is as expressed in Equation (36). The result is as shown in Figure 17. The resulting standard deviation is from 0.1663°/s to 0.0638°/s.

(36) $$\begin{array}{cc} \Omega_{SF2} & =\Omega_{SF1}\cdot \left({a^{\prime \prime }}_{1}\cdot T^{2}+{a^{\prime \prime }}_{2}\cdot T+{a^{\prime \prime }}_{3}\right)\\ & =\Omega_{SF1}\cdot \left(\text{-1.26e-07}\cdot T^{2}\text{-1}{\text{.83e-05}}_{2}\cdot T+0.9979\right) \end{array}$$

### 4.4. Noise Filter

**Figure 18:**
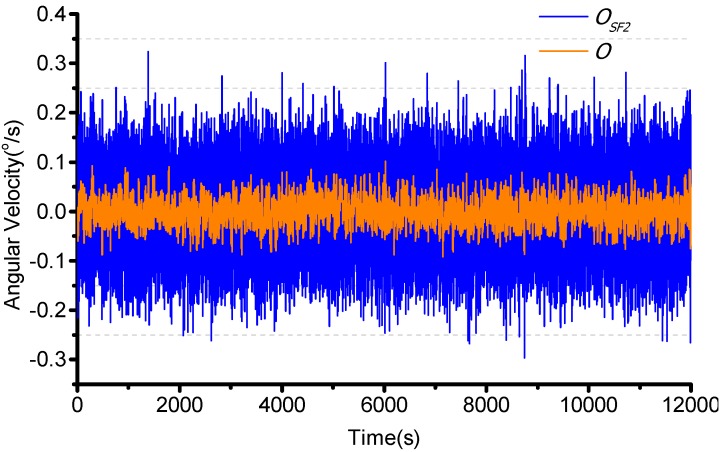
Noise Filter.

**Figure 19:**
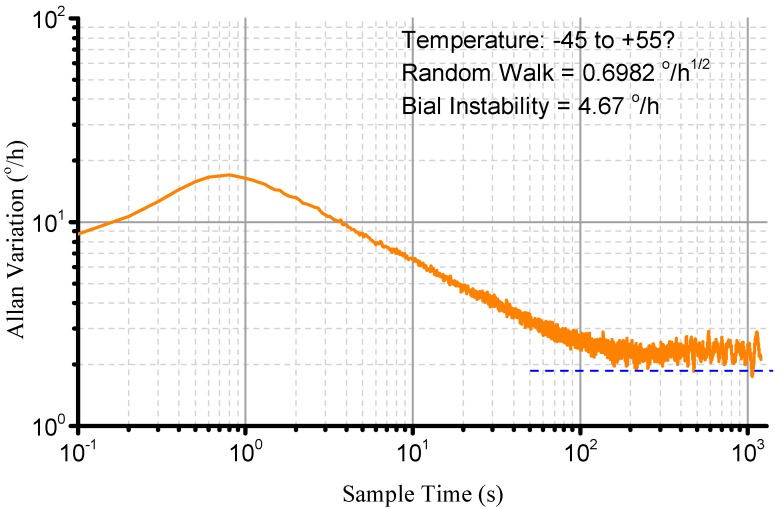
Allan Variance.

Finally, the noise is presented using the FIR filter. The cutoff frequency is 200 Hz and a 50 order filter is used. The temperature box is set from −45 °C to +55 °C and turntable is stationary. The result is as shown in Figure 18. The signal performance is then evaluated using Allan variance as shown in Figure 19. It is shown that the bias instability is 4.67°/h (1 δ) and the random walk is 0.6982°/h^1/2^.

## 5. Conclusions

This paper focuses on error modeling and compensation of the BVG. The dynamic equation is firstly established based on a study of BVG working mechanism. This equation is used to evaluate the relationship between the angular rate output signal and the bell-shaped resonator characteristics, the influence of the main error sources is evaluated and an error model of BVG is set up. The error sources are classified based on their error propagation properties, and the compensation method is presented based on the error model. Finally, the error model and compensation method are used to experimentally calibrate the BVG, which includes rough compensation, temperature and bias compensation, scale factor compensation and noise filter. The experimentally obtained bias instability is from 20.5°/h to 4.67°/h, the random walk is from 2.7821°/h^1/2^ to 0.6982°/h^1/2^ and the nonlinearity is from 0.2% to 0.03%. Based on the error compensation, it is shown that there is a good linear relationship between the sensing signal and the angular velocity, suggesting that the BVG is a good candidate for the field of low and medium rotation speed measurements.

However, the performance of BVG is lower than HRG and CVG in areas such as bias instability and randomness and BVG is a medium/low performance gyro and has a wide gap to the micro optical gyro. However, we will pay close attention to these technologies. In the future, we will study how to improve performance. In addition to this, we will study more deeply the mathematic model of the bell-shaped resonator and consider using non-contact types to design this gyroscope.
